# Exposure to road traffic and railway noise and associations with blood pressure and self-reported hypertension: a cohort study

**DOI:** 10.1186/1476-069X-10-92

**Published:** 2011-10-28

**Authors:** Mette Sørensen, Martin Hvidberg, Barbara Hoffmann, Zorana J Andersen, Rikke B Nordsborg, Kenneth G Lillelund, Jørgen Jakobsen, Anne Tjønneland, Kim Overvad, Ole Raaschou-Nielsen

**Affiliations:** 1 Institute of Cancer Epidemiology, Danish Cancer Society, Copenhagen, Denmark; 2 National Environmental Research Institute, Aarhus University, Roskilde, Denmark; 3 IUF-Leibniz Research Institute for Environmental Medicine and Medical Faculty, Heinrich Heine University of Düsseldorf, Düsseldorf, Germany; 4 Rambøll Danmark A/S, Aarhus, Denmark; 5 Danish Environmental Protection Agency, Copenhagen, Denmark; 6 Department of Epidemiology, School of Public Health, Aarhus University, Aarhus, Denmark; 7 Department of Cardiology, Centre for Cardiovascular Research, Aalborg Hospital, Aarhus University Hospital, Aalborg, Denmark

**Keywords:** blood pressure, transport noise, road traffic, railway, epidemiology, cohort

## Abstract

**Background:**

Epidemiological studies suggest that long-term exposure to transport noise increases the risk for cardiovascular disorders. The effect of transport noise on blood pressure and hypertension is uncertain.

**Methods:**

In 1993-1997, 57,053 participants aged 50-64 year were enrolled in a population-based cohort study. At enrolment, systolic and diastolic blood pressure was measured. Incident hypertension during a mean follow-up of 5.3 years was assessed by questionnaire. Residential long-term road traffic noise (L_den_) was estimated for 1- and 5-year periods preceding enrolment and preceding diagnosis of hypertension. Residential exposure to railway noise was estimated at enrolment. We conducted a cross-sectional analysis of associations between road traffic and railway noise and blood pressure at enrolment with linear regression, adjusting for long-term air pollution, meteorology and potential lifestyle confounders (N = 44,083). Incident self-reported hypertension was analyzed with Cox regression, adjusting for long-term air pollution and potential lifestyle confounders.

**Results:**

We found a 0.26 mm Hg higher systolic blood pressure (95% confidence intervals (CI): -0.11; 0.63) per 10 dB(A) increase in 1-year mean road traffic noise levels, with stronger associations in men (0.59 mm Hg (CI: 0.13; 1.05) per 10 dB(A)) and older participants (0.65 mm Hg (0.08; 1.22) per 10 dB(A)). Road traffic noise was not associated with diastolic blood pressure or hypertension. Exposure to railway noise above 60 dB was associated with 8% higher risk for hypertension (95% CI: -2%; 19%, P = 0.11).

**Conclusions:**

While exposure to road traffic noise was associated with systolic blood pressure in subgroups, we were not able to identify associations with hypertension.

## Background

Increasing noise from traffic occurs in parallel with industrialization and urbanization. Exposure to noise can interfere with relaxation and concentration and during the night, noise exposure at normal urban levels has been associated with sleep disturbances [1,2]. This is believed to result in a stress reaction with activation of the sympathetic and endocrine system, which can lead to changes in blood pressure (BP), heart rate and release of stress hormones [3-5].

An overview from 2006 of the effects of exposure to transport noise (road, air and rail) on cardiovascular health concluded that transport noise is associated with hypertension [6], which has mostly been verified by later studies on road traffic noise [7-13], whereas the two studies investigating railway noise are inconclusive [11,13]. The majority of the studies on traffic noise and hypertension are cross-sectional and no studies have prospectively investigated the association between road traffic noise and the incidence of hypertension. The overview from 2006 found no consistent associations between transport noise and the systolic and diastolic BP, which might be explained by the fact that the studies conducted so far suffered from insufficient power and narrow exposure ranges [6].

Railway noise has generally been considered as the least annoying transportation source [1], and, therefore, it has not achieved the same attention as road traffic and airport noise. However, recent studies suggest that nocturnal railway noise is as disturbing to sleep as road traffic noise [14,15] and that railway noise has important short-term impact on the cardiovascular system [16] and might increase the risk for hypertension [13].

The few studies that have investigated effects of exposure to long-term air pollution on BP and hypertension has found air pollution to be positively associated with systolic and diastolic BP as well as with hypertension [17-19]. Although exposure to road traffic noise and air pollution have been found to correlate and both are suspected of affecting BP, only two studies on hypertension has included both exposures in the same model [7,20]. The results suggest that exposure to road traffic noise is associated with a higher risk for hypertension both before and after adjustment for air pollution.

In this study we tested the hypothesis that long-term exposure to road traffic and railway noise increase systolic and diastolic BP as well as the risk for hypertension, taking exposure to air pollution into account.

## Methods

### Study population

The study was based on the Diet, Cancer and Health cohort where 160,725 randomly chosen subjects, living in the Copenhagen or Aarhus area, were invited to participate between 1993 and 1997. They were a random sample of all eligible cancer free subjects between 50-64 years of age at time of invitation [21]. All in all 57,053 subjects accepted the invitation and were enrolled into the cohort. A follow-up survey of all eligible cohort participants (excluding dead or emigrated participants) was conducted in 2000-2002. In total, 54,379 participants (96%) received an invitation and a follow-up questionnaire by mail, including questions on health status. The response rate was 83.3%, corresponding to 45,271 participants. Participation was based on written informed consent. The study was conducted in accordance with the Helsinki Declaration and approved by the local ethical committees.

At enrolment, each participant completed self-administered questionnaires including questions on lifestyle habits (such as questions on tobacco smoking, alcohol intake and sport during leisure time) and health status, such as whether they suffered or ever had suffered from hypertension, and whether they received or had ever received medication for hypertension. Height and weight were measured according to standardized protocols.

### Exposure assessment

The level of road traffic noise was modeled at each address at which the cohort members had lived from five years preceding enrolment until follow-up in 2000-2002 by use of SoundPLAN (version 6.5, http://www.soundplan.dk/). This noise calculation program implements the joint Nordic prediction method for road traffic noise, which has been the standard method for noise calculation in Scandinavia during many years [22,23].

The input variables for the noise model were: 1) geographical coordinates and height of each dwelling over terrain, 2) road lines with information on yearly average daily traffic, traffic composition, traffic speed and road type (motorway, rural highway, road wider than 6 m, and other road), 3) building polygons for all buildings including information on building height (to include screening from buildings). We assumed that the terrain was flat, which is a reasonable assumption in Denmark, and that urban areas, roads, and areas with water were hard surfaces whereas all other areas were acoustically porous. No information was available on noise barriers.

Road traffic noise was calculated as the A-weighted level (L_Aeq_) at the most exposed façade of the dwelling at each address for the day (07:00-19:00 h), evening (19:00-22:00 h) and night (22:00-07:00 h) and expressed as L_den _by applying a 5 dB(A) penalty for the evening and a 10 dB(A) penalty for the night [24]. The noise model used is very similar to the model recommended by the EU directive in most points, except small corrections to better suit Danish conditions including calculating noise in level with the dwelling (EU: 4 m above ground) and use of the evening period 19:00-22:00 h (EU: 19:00-23:00 h).

Exposure to railway noise was calculated as the A-weighted equivalent level (L_Aeq,24 h_) at enrolment with the joint Nordic prediction method for railway noise based on information about traffic in 1993-2000. The model calculated exposures in the range of 60 to 80 dB. Screening by designated noise screens and buildings was not considered. The estimated level of railway noise is assumed to be representative for the whole study period (1990 - 2002), as neither high speed rail tracks nor other new rail tracks have been opened in Denmark during the period, and moreover, cargo rail traffic has been stable. The noise impact from airports and airfields was determined from information about noise zones obtained from local environmental authorities. The programs DANSIM and INM3, which fulfill the joint Nordic criteria for air traffic noise calculations, were used. The noise curves for railway and aircraft noise were transformed into digital maps, and noise levels were linked to each address by geocodes. Only very few of the included cohort members (<1%) were exposed to aircraft noise of 55 dB(A) or more, and as this study, therefore, is not powered to investigate effects of air traffic noise these persons were excluded from the dataset.

The yearly average concentration of nitrogen oxides (NO_x_) in the air was modeled at each address at which the cohort members had lived from five years prior to enrolment until follow-up in 2000-2002 by the Danish AirGIS modeling system. AirGIS allows calculation of air pollution at a location as the sum of 1) the local air pollution from traffic in the streets with the Operational Street Pollution Model, 2) the urban background contribution calculated with a simplified area source dispersion model, and 3) a regional background contribution [25]. Input data for the AirGIS system included traffic data for the period 1988-2000, emission factors for the Danish car fleet, street and building geometry, building height and meteorological data [26]. The AirGIS system has been successfully validated in several studies [25-27]. We used NO_x _as indicator for air pollution from traffic because measured NO_x _has been found to correlate strongly with other traffic-related pollutants in Danish streets such as particles: r = 0.93 for total particle number concentration (10-700 nm) and r = 0.70 for PM_10 _[28,29].

### Blood pressure measurement

At enrolment trained staff members measured brachial artery BP by automated TAKEDA UA 751 or UA 743 Oscillometers once. The measurement was conducted in the supine position after a minimum of 5 minutes rest and at least 30 minutes after tobacco smoking and intake of food, tea or coffee. If systolic BP was 160 mm Hg or more, or diastolic BP was 95 mm Hg or more, the measurement was repeated after an interval of at least 3 min and the lowest measurement of the two was used. We excluded all participants which in the enrolment questionnaire answered that they received or had received medication for hypertension.

### Incidence of hypertension

Information on hypertension was assessed by questionnaire at enrolment (1993-1997) and in the follow-up survey (2000-2002), including questions whether a medical doctor had ever diagnosed the participants with hypertension, the age (in whole number) at which they were diagnosed with hypertension and whether they received or had ever received antihypertensive medication. All participants reporting to have hypertension in the enrolment questionnaire were excluded from the incidence analyses. Participant reporting to have been diagnosed with hypertension between enrolment and follow-up were censored at the age they reported to have been diagnosed.

### Statistical methods

#### Cross-sectional analysis of systolic and diastolic BP

We used mixed linear models to test for associations between residential exposure to long-term road traffic, L_den _(continuous), and exposure to railway noise, L_Aeq,24 _(categorical), and systolic and diastolic BP (PROC MIXED, SAS 9.1, SAS-Institute, Inc., USA), with centre of enrolment (Copenhagen or Aarhus) as a random effect. We included centre as random effect as the BP measurements generally were higher in the Copenhagen centre than in Aarhus centre, which might be explained by differences in procedures etc. between the two centers.

We conducted analyses adjusted for potential confounders in two steps: 1) age (linear) and gender; 2) further adjustment for calendar-year; area of residence (Copenhagen city, Aarhus city and surroundings of Copenhagen/Aarhus (7-25 km from city centre, sub-urban or rural)); length of school attendance (<8, 8-10, >10 years); socioeconomic status (SES) of municipalities or district for Copenhagen municipality at enrolment (Copenhagen is the largest Danish municipality and was split into 10 districts with regard to SES; average number of citizens in the municipalities/districts was 43,000) in three groups (low, medium and high SES) based on baseline municipality/district information on education, work market affiliation and income; body mass index (BMI, kg/m^2^, linear); smoking status (never, former, current); alcohol intake (yes/no; g/day among drinkers, linear); sport during leisure time (yes/no; h/week among active, linear); air pollution (NO_x _, μg/m^3^, mean time-weighted average exposure periods 1- and 5- year preceding BP measurement); season (winter, spring, summer and autumn); and mean of ambient temperature and humidity the three days preceding the BP measurement. As an alternative way of modeling, we categorized the exposure into three groups using the 50^th ^and 90^th ^percentiles as cut-points. We conducted two sensitivity analyses; 1) only participants with normal BP were included (systolic BP ≤ 140 and/or diastolic BP ≤ 90, N = 25,248) and 2) subjects on antihypertensive medication were included to the study base resulting in a total population of 50,315 participants.

In exploratory analyses, we tested for interactions between modeled long-term exposure to road traffic noise (1-year) and gender, age, years of education, SES, temperature (above and below 15°C) and diagnosis of cardiovascular disease before enrolment.

#### Follow-up for hypertension

Incident hypertension was analyzed with a Cox proportional hazards model with age as the underlying time [30]. We used left truncation at age of enrolment, so that subjects were considered at risk from enrolment into the cohort, and right censoring at age of event (self-reported hypertension) or age at follow-up survey, whichever came first. All analyses were stratified by gender and calendar-year. Exposure to long-term air pollution was modeled as time-dependent variables using the long-term time-weighted average NO_x _concentrations (1- and 5-year means) at a given age.

The incidence rate ratios (IRRs) for hypertension in association with modeled long-term road traffic noise was calculated using the preceding 1- and 5-year mean road traffic noise exposure (continuous) at the time of diagnosis (event) compared with the preceding 1- and 5-year mean road traffic noise exposure of all cohort members at risk at that point in time where they had the same age as the event person. Also, IRRs for hypertension in association with exposure to railway noise at baseline above 60 dB was calculated. We conducted analyses adjusted for a priori defined confounders in two steps: 1) gender; 2) further adjustment for baseline information on smoking status, length of school attendance, alcohol intake, BMI, sport during leisure time, SES, area, calendar-year and air pollution.

#### Linearity

The assumption of linearity of the associations between both road traffic noise (measured in decibel) and the covariates (air pollution, age, BMI, alcohol intake, sport during leisure time and ambient temperature and humidity) and health outcomes (BP and hypertension) was evaluated both visually and by formal testing with linear spline models with boundaries placed at the nine deciles for the included cohort members (systolic and diastolic BP) or cases (hypertension) [31]. We found no deviation from linearity for road traffic noise in relation to the health outcomes. For air pollution the association between the exposure variables and the health outcomes did not deviated from linearity after transformation by the logarithm and for ambient temperature the association did not deviate after adding a squared term. The remaining covariates did not deviate from linearity.

## Results

### Systolic and diastolic BP

Out of 57,053 subjects, we excluded 571 with cancer diagnoses before baseline, 2,737 with incomplete residential address information, 63 without BP measurement, 2,961 with missing information on covariates, 6,285 with hypertensive medicine at or prior to enrolment and 353 exposed to more than 55 dB(A) residential aircraft noise, leaving 44,083 participants.

Table 1 shows the distribution of baseline characteristics according to exposure above and below 55 dB(A) 1-year road traffic noise at enrolment among the 44,083 cohort participants in the cross-sectional BP study. Participants living at residences with low road traffic noise exposure tended to have higher SES, to smoke less, to be more physically active and to be exposed to lower levels of air pollution and railway noise than participants living in high exposed areas.

**Table 1:** Baseline characteristics according to exposure to road traffic noise above and below 55 dB(A) (L_den_) at enrolment of 44,083 cohort participants

	Mean L_den _levels the year preceding enrolment			
	
Characteristic, at enrolment	< 55 dB(A)			≥ 55 dB(A)
		
	% (N)	Median (5-95 percentiles)	% (N)	Median (5-95 percentiles)
All	100 (18,522)		100 (25,561)	

Age (years)		55.7 (50.7-64.0)		56.1 (50.7-64.2)

Gender				

Women	51 (9,372)		53 (13,556)	

Men	49 (9,150)		47 (12,005)	

Years of education				

≤ 7	30 (5,609)		34 (8,772)	

8-10	46 (8,557)		46 (11,682)	

≥ 11	24 (4,356)		20 (5,107)	

Municipality SES^a^				

Low	9 (1,638)		18 (4,630)	

Medium	70 (13,028)		61 (15,544)	

High	21 (3,856)		21 (5,387)	

Area				

Copenhagen city	16 (3,013)		33 (8,473)	

Aarhus city	44 (8,091)		19 (4,937)	

Copenhagen/Aarhus surroundings	40 (7,418)		48 (12,151)	

BMI (kg/m^2^)		25.2 (20.4-32.4)		25.4 (20.3-33.0)

Smoking				

Never	38 (7,039)		34 (8,648)	

Former	29 (5,284)		27 (6,865)	

Current	33 (6,199)		40 (10,048)	

Drinking alcohol				

No	2 (347)		2 (607)	

Yes	98 (18,175)		98 (24,954)	

Among active drinkers (g/day)		13.4 (1.31-61.2)		13.4 (1.03-66.9)

Physical activity				

No	42 (7,789)		47 (11,990)	

Yes	58 (10,733)		53 (13,571)	

Among active (h/week)		2.0 (0.5-7.0)		2.0 (0.5-7.0)

Railway noise ≥ 60 dB	13 (2,364)		19 (4,885)	

L_den_, 1 year^b ^(dB(A))		51.7 (46.3-54.7)		61.1 (55.5-71.7)

NO_x_, 1-year^b ^(μg/m^3^)		17.5 (13.8-25.3)		25.3 (15.9-112)

^a ^Socioeconomic status of municipalities based on municipality information on education, work market affiliation and income

^b ^Time-weighted average level at residences for one years preceding enrolment

The distributions of systolic and diastolic BP were slightly right-skewed. However, similar results were observed for untransformed and log-transformed values and regression estimates for the untransformed data are presented. The Spearman correlation between road traffic noise and NO_x _was 0.69 and 0.70 for 1- and 5-year means, respectively, and 0.97 between 1- and 5-years of exposure to road traffic noise.

Associations between road traffic noise and the systolic and diastolic BP are shown in Table 2. In the categorical analyses the highest exposure group (10% highest exposed) had a 0.79 mm Hg (95% CI: -0.04; 1.62) and a 0.85 mm Hg (95% CI: 0.02; 1.67) higher systolic BP compared with the lowest exposure group for 1-year and 5-mean, respectively. The linear analyses showed a 0.26 (95% CI: -0.11; 0.63) mm Hg higher level of systolic BP per 10 dB(A) higher level of road traffic noise (1-year mean). No associations between road traffic noise and diastolic BP were observed.

**Table 2:** Associations between road traffic noise and the systolic and diastolic blood pressure at enrolment

		Differences in systolic blood pressure (mm Hg) (95% CI)			Differences in diastolic blood pressure (mm Hg) (95% CI)	
			
Road traffic noise (L_den_)	N	Adjusted by age and gender	Adjusted by age, gender, calendar-year, area, lifestyle factors^a ^, air pollution^b^, season, temperature and humidity	P	Adjusted by age, gender, calendar-year, area, lifestyle factors^a ^, air pollution^b^, season, temperature and humidity	P
1-year mean (dB(A))^c^						

< 56.4	22,041	0 (ref)	0 (ref)		0 (ref)	

56.4-67.3	17,635	0.12 (-0.27;0.50)	0.17 (-0.23; 0.58)	0.41	-0.00 (-0.22; 0.21)	0.98

> 67.3	4,407	0.23 (-0.39; 0.85)	0.79 (-0.04; 1.62)	0.06	0.11 (-0.33; 0.55)	0.62

*Linear trend per 10 dB(A)*	*44,083*	*0.09 (-0.18; 0.37)*	*0.26 (-0.11; 0.63)*	*0.17*	*0.07 (-0.13; 0.27)*	*0.50*

						

5-year mean (dB(A))^c^						

< 56.3	22,041	0 (ref)	0 (ref)		0 (ref)	

56.3-67.3	17,635	0.04 (-0.35; 0.42)	0.08 (-0.33; 0.48)	0.71	-0.02 (-0.23; 0.20)	0.88

>67.3	4,407	0.40 (-0.23; 1.02)	0.85 (0.02; 1.67)	0.04	0.12 (-0.32; 0.55)	0.60

*Linear trend per 10 dB(A)*	*44,083*	*0.07 (-0.21; 0.35)*	*0.14 (-0.24; 0.53)*	*0.47*	*0.03 (-0.18; 0.23)*	*0.78*

All analyses include centre of enrolment as random factor

^a ^Lifestyle factors: smoking, BMI, length of school attendance, municipality SES, alcohol intake and physical activity.

^b ^Time-weighted average concentration of NO_x _at residences 1 and 5 years preceding enrolment

^c ^The cut-off points between exposure groups were the 50^th ^and 90^th ^percentiles for the participants at the time of enrolment into the cohort

Associations between road traffic noise and BP seemed to be modified by gender with only effect among men (0.59 mm Hg per 10 dB(A); 95% CI: 0.13; 1.05), by age with only effect among participants above 60 years of age (0.65 mm Hg per 10 dB(A); 95% CI: 0.08; 1.22) and by outdoor temperature with only effect at outdoor temperatures above 15°C (0.87 mm Hg per 10 dB(A); 95% CI: 0.07; 1.66) (Table 3). There seemed to be no effect modification by education or SES.

**Table 3:** Modification of associations between the preceding 1-year L_den _and systolic blood pressure by baseline characteristics

Covariates	N	Differences in systolic blood pressure (mm Hg) (95% CI) per 10 dB(A) increase in 1-year exposure to road traffic noise	P	P_interaction_
Gender				0.01

Women	22,928	-0.04 (-0.48; 0.40)	0.87	

Men	21,155	0.59 (0.13; 1.05)	0.01	

Age				0.08

≤ 60 years	32,962	0.13 (-0.28; 0.53)	0.54	

> 60 years	11,121	0.65 (0.08; 1.22)	0.03	

Years of education				0.37

≤ 7	14,381	0.27 (-0.25; 0.78)	0.31	

8-10	20,239	0.40 (-0.06; 0.87)	0.09	

≥ 11	9,463	-0.08 (-0.70; 0.54)	0.80	

Municipality SES^a^				0.63

Low	6,268	0.57 (-0.22; 1.36)	0.16	

Medium	28,572	0.19 (-0.23; 0.61)	0.37	

High	9,243	0.32 (-0.32; 0.95)	0.33	

Outdoor temperature (°C)				0.09

≤ 15	38,894	0.18 (-0.20; 0.57)	0.35	

> 15	5,189	0.87 (0.07; 1.66)	0.03	

Prior diagnosis of cardiovascular disease^b^				0.29

Yes	998	1.15 (-0.54; 2.85)	0.18	

No	43,085	0.24 (-0.13; 0.62)	0.21	

Analyses adjusted by age, gender, calendar-year, area, lifestyle factors (smoking, BMI, length of school attendance, municipality SES, alcohol intake and physical activity), air pollution season, temperature and humidity

^a ^Socioeconomic status of municipalities based on municipality information on education, work market affiliation and income

^b ^A diagnosis of myocardial infarction and/or stroke before enrolment

In a sensitivity analysis including only participants with normal BP, the highest exposure group (10% highest exposed) had a 0.07 mm Hg (95% CI: -0.54; 0.69) higher systolic BP compared with the lowest exposure group (1-year mean). In another sensitivity analysis including subjects on antihypertensive medication, resulting in a total population of 50,315 participants, the highest exposure group (10% highest exposed) had a 0.90 mm Hg (95% CI: 0.10; 1.71) higher systolic BP compared with the lowest exposure group (1-year mean).

Exposure to railway noise at levels between 60 and 70 dB was associated with a 0.03 mm Hg higher systolic BP (95% CI -0.44; 0.51, N = 6,886), and levels above 70 dB was associated with a 0.84 mm Hg (95% CI: -1.05; 2.74, P = 0.38) higher systolic BP (N = 363) as compared with the reference group (less than 60 dB). Similarly, the estimates for diastolic BP were -0.03 mm Hg (95% CI: -0.29; 0.22) and 0.62 mm Hg (95% CI: -0.39; 1.62) in the medium and high exposure group, respectively.

### Hypertension

Out of the 45,271 persons that filled in the follow-up questionnaire we excluded 7110 with hypertension at or prior to enrolment, 1841 participants with missing or contradictory answers to the hypertension questions given at enrolment and follow-up, 2897 with incomplete residential address information, 148 with missing information on covariates, and 640 exposed to more than 55 dB(A) residential aircraft noise during the follow-up period, leaving a study base of 32,635 participants. Among these, 3145 participants reported that they had been diagnosed with hypertension within the follow-up period.

The distribution of baseline characteristic according to exposure to road traffic noise among the 32,635 participants followed up for hypertension was very similar to the distributions shown in Table 1 (results not shown).

We found no associations between exposure to long-term road traffic noise and risk for self-reported hypertension in the subset of participants, who responded at the follow-up survey (Table 4). There were no effect modifications by categories of road traffic noise, gender, age, education, SES or prior diagnosis of cardiovascular disease.

**Table 4:** Incidence rate ratios (IRRs) per 10 dB(A) increase in road traffic noise and for railway noise above 60 dB in relation to self-reported hypertension

		Road traffic noise, 1-y mean			Road traffic noise, 5-y mean		Railway noise ≥ 60 dB, enrolment	
		
		IRR (95% CI)			IRR (95% CI)		IRR (95% CI)	
				
	N cases	Adjusted by age and gender	Adjusted by age, gender, calendar-year, area, lifestyle factors^a^, air pollution^b ^and railway noise	**P ^c^**	Adjusted by age, gender, calendar-year, area, lifestyle factors^a^, air pollution^b ^and railway noise	**P ^c^**	Adjusted by age, gender, calendar-year, area, lifestyle factors^a^, air pollution^b ^and road traffic noise	**P ^c^**
All	3145	1.02 (0.97-1.08)	1.02 (0.95-1.10)		0.97 (0.90-1.05)		1.08 (0.98-1.19)	

Noise (L_den_, dB(A))								

< 55	1237	1.09 (0.87-1.37)	1.10 (0.87-1.38)	0.36	1.03 (0.81-1.30)	0.31	-	

55-65	1410	0.94 (0.78-1.13)	1.04 (0.77-1.12)		0.90 (0.74-1.09)		-	

> 65	498	1.16 (0.88-1.53)	1.15 (0.86-1.54)		1.17 (0.86-1.58)		-	

Gender								

Women	1668	1.02 (0.95-1.10)	1.02 (0.94-1.12)	0.89	0.97 (0.89-1.07)	0.99	1.10 (0.97-1.25)	0.70

Men	1477	1.02 (0.95-1.10)	1.02 (0.92-1.12)		0.97 (0.88-1.07)		1.06 (0.92-1.22)	

Age (years)								

≤ 60	1571	1.04 (0.96-1.11)	1.03 (0.94-1.13)	0.58	0.98 (0.89-1.08)	0.85	1.07 (0.94-1.22)	0.86

> 60	1574	1.01 (0.93-1.08)	1.00 (0.92-1.10)		0.97 (0.88-1.06)		1.09 (0.95-1.24)	

Years of education								

≤ 7	1060	1.06 (0.97-1.16)	1.06 (0.96-1.18)	0.51	1.02 (0.91-1.13)	0.43	1.13 (0.97-1.32)	0.62

8-10	1483	0.99 (0.92-1.08)	1.01 (0.92-1.10)		0.96 (0.87-1.06)		1.08 (0.95-1.24)	

≥ 11	602	0.98 (0.86-1.10)	0.98 (0.86-1.12)		0.92 (0.80-1.06)		0.99 (0.78-1.24)	

Municipality SES^a^								

Low	461	0.95 (0.83-1.09)	0.98 (0.84-1.14)	0.66	0.93 (0.79-1.09)	0.78	1.08 (0.88-1.32)	0.95

Medium	2039	1.04 (0.97-1.11)	1.03 (0.95-1.13)		0.98 (0.90-1.07)		1.07 (0.94-1.21)	

High	645	1.01 (0.89-1.14)	0.99 (0.87-1.13)		0.96 (0.84-1.10)		1.11 (0.91-1.36)	

Prior diagnosis of cardiovascular disease^b^								

Yes	51	1.22 (0.82-1.82)	1.22 (0.81-1.84)	0.37	1.11 (0.73-1.69)	0.52	0.74 (0.35-1.58)	0.33

No	3094	1.02 (0.97-1.07)	1.02 (0.94-1.10)		0.97 (0.90-1.05)		1.09 (0.99-1.20)	

^a ^Lifestyle factors at enrolment: smoking, BMI, length of school attendance, municipality SES, alcohol intake and physical activity.

^b ^Time-weighted average concentration of NO_x _at residences 1 and 5 years preceding enrolment

^c ^P for interaction

Exposure to railway noise at enrolment of 60 dB or more was associated with an 8% higher risk for hypertension (95% CI: -2%; 19%, P = 0.11) as compared with cohort participants exposed to less than 60 dB (Table 4). There was no effect modification by gender, age, education, SES or prior diagnosis of cardiovascular disease.

## Discussion

Our study showed that long-term exposure to road traffic noise was weakly associated with a higher systolic BP in a cross-sectional design, whereas long-term exposure to road traffic noise was not associated with risk for self-reported development of hypertension in a prospective design. Exposure to railway noise of 60 dB or more was associated with an 8% higher risk for hypertension.

### Strengths and weaknesses

Strengths of both the cross-sectional and the prospective study included the large study population, with detailed information on various potential confounders. Furthermore, access to residential address histories was a major advantage in estimation of long-term exposure to both road traffic noise and air pollution. As one of the first studies ever within this area we adjusted for exposure to long-term air pollution. Air pollution is a potentially important confounder in our study because residential exposure to road traffic noise and air pollution is known to correlate [7,32], and exposure to traffic-related air pollution might affect BP and hypertension, though the results are inconclusive [17,33-35].

Although the Nordic Prediction method for road traffic and railway noise has been used in many years, such estimation of noise is inevitably associated with some degree of uncertainty, e.g. because of inaccurate input data, which would result in exposure misclassification. Furthermore, we did not have information on noise barriers for road traffic noise and neither noise barriers nor screening by buildings for railway noise. However, as the models does not distinguish between participants with 'high' or 'low' BP/risk of hypertension such misclassification is believed to be non-differential and influence the risk estimates towards the neutral value. Also, we have previously found that modeled road traffic noise was positively associated with the risk for stroke in the same cohort [32], which speaks in favor of the modeled values.

A limitation is that we only had information on residential addresses. We believe that such imprecision in the noise exposure assessment is similar between participants with 'high' and 'low' BP and may, therefore, have attenuated the risk estimates. Neither did we have information on bedroom location, window opening habits, neighbor-noise and hearing impairment, which might all influence the noise exposure. However, studies have found the effect of noise to be stronger when these factor are considered [6,36], suggesting that the effect of noise might be underestimated in the present study.

A limitation of the BP part of this study is the cross-sectional design with no repeated measures for the participants. The BP measure is variable and depends on many different factors such as age, gender and temperature. Though we have adjusted for many of these possible confounders, the results should be replicated in a design with repeated measures before firm conclusions can be made.

The measurement of systolic and diastolic BP in our study was standardized but not in accordance with standard recommendations for diagnosing hypertension where several measurements of BP are requested. If the systolic BP was 160 mm Hg or more or the diastolic BP was 95 mm Hg or more, the measurement was repeated and only the lowest measurement was registered. This has most likely resulted in a systematic bias towards lower values in participants with high BP, which could have biased the BP estimate towards the neutral value. A sensitivity analysis, with restriction of the sample to participants with normal BP values, and, thus, the participants least likely to have repeated BP measurement, showed no effects of road traffic noise on BP. However, this sensitivity analysis may introduce selection bias by limiting the analysis to possibly non-susceptible participants.

We excluded participants reporting that they used anti-hypertensive medication, because the medication might prevent or reduce an effect of noise on BP. A sensitivity analysis including participants with hypertension at enrolment, showed that the BP estimates in the highest exposure group were slightly higher than the estimates obtained in the primary analysis, suggesting that we might have excluded the most susceptible participants.

Our prospective study of hypertension has some limitations. First, all information on hypertension is self-reported and, thus, the actual number of hypertensive participants is probably underestimated, due to the fact that hypertension is usually symptom-free, and, therefore, a part of the participants will have un-diagnosed hypertension and classify themselves as having normal BP. Therefore, a number of participants which were actually hypertensive at baseline were falsely included as non-hypertensive. Such misclassification may have lead to a systematic bias, e.g. if underreporting was most prominent in low SES groups, who are often exposed to the highest levels of transport noise, this could have influenced the risk estimates towards the neutral value. The analysis of hypertension is based on those respondents who have survived and responded at follow up. Participants who died (and presumably had a higher risk of hypertension) are not included, which could have influenced the risk estimates towards the neutral value. Also, we excluded participants who were likely to use antihypertensive treatment at enrolment, and thus the participants who were most susceptible to exposure to noise, which might have lead to an underestimation of the association between noise and risk for hypertension. Also, the use of hypertension as a dichotomous outcome results in loss of information, which makes this endpoint fairly insensitive to small effects of noise.

### Systolic and diastolic BP

High blood pressure is a major risk factor for cardiovascular disease and, therefore, even small increases in BP from road traffic noise may have high impact on public health [37]. Earlier studies on transport noise and BP are inconclusive, as both positive and negative associations have been indicated [38,39]. A recent overview concluded that there was no evidence that transport noise increased the systolic and diastolic BP in the adult population, but that the hypothesis of an effect of noise on BP could not be discarded as the studies conducted so far suffered from insufficient power and narrow exposure ranges [6]. Our study is more than 10 times larger than previous studies on transport noise and BP, and with its more than 44,000 participants sufficiently powered to detect even small differences in BP. Furthermore, the study has a relatively large exposure span (36 to 82 dB(A) for road traffic noise), especially considering that a 3 dB(A) increase corresponds to a doubling in the acoustical energy.

Overall, our study suggested that long-term exposure to road traffic noise was only weakly and mainly insignificantly associated with a higher systolic BP, which confirms previous studies indicating that the association between traffic noise and BP/hypertension is rather weak [6-13]. However, our findings indicated that certain groups of people might be susceptible groups in relation effects of road traffic noise on BP. One possible susceptible group is men as our results suggest that the association between road traffic noise and systolic BP was present only among men. Other studies have investigated effect modification by gender in relation to the association between traffic noise and BP and hypertension, but the results are inconclusive, as some studies suggests no difference [7,10], some suggest strongest association among men [9,11,20] whereas others find strongest associations among women [8,40]. More studies are needed.

Older people might be another susceptible group with regard to the hazardous effects of traffic noise as the relationship between exposure to road traffic noise and BP was found to be strongest among the older participants (over 60 years). Sleep disturbances contribute to cardiovascular risk [41,42], and it is therefore believed that night noise exposure is more harmful than daytime exposure [43]. The sleep structure generally becomes more fragmented with age and older people are, thus, more susceptible to sleep disturbances [44,45]. This could explain why exposure to road traffic noise was mainly related to high BP among the oldest cohort participants in our study. Unfortunately we could not further investigate if night-time noise was stronger associated with BP than L_den_, as exposure to road traffic noise during the night (L_n_) was highly correlated with L_den _and, therefore, we could not separate their effects.

We found that the effect of road traffic noise was strongest at temperatures above 15 °C. A possible explanation is that during warm periods many people sleep with their windows open which could result in higher levels of traffic noise in the bedroom. Unfortunately, we do not have any information on whether people sleep with open windows in this study.

Our results suggest that exposure to railway noise above 70 dB might increase the systolic BP. Similarly, the only other study that has previously investigated the association between railway noise and BP found that exposure to railway noise significantly increased the systolic BP [13]. However, since only 363 persons were exposed to railway noise above 70 dB in the present study and since the increase in systolic BP was clearly insignificant, this finding might be a chance finding.

### Hypertension

Many cross-sectional studies have investigated the relationship between exposure to road traffic noise and hypertension and/or use of hypertension medication, and the overall picture from these studies is that road traffic noise results in a slightly higher prevalence of hypertension [7-13]. Our study is the first prospective study to examine the association between road and railway traffic noise and the incidence of hypertension. We found no overall association between exposure to road traffic noise and self-reported hypertension, as well as no effects in potentially susceptible groups such as older people and people with cardiovascular disease. Considering the limitations of using self-reports of hypertension more studies with validated diagnoses of hypertension are needed before any conclusions can be made.

Two previous cross-sectional study have investigated effect of railway noise on the prevalence of hypertension and one found a significant positive association [13] whereas the other study found no associations [11]. We found that railway noise seemed to be weakly associated with the risk for hypertension, supported by recent studies suggesting that short-term railway noise has important impact on the cardiovascular system, such as the heart rate response [16], though this impact has been found to habituate with long-term exposure [46]. However, all our findings on railway noise are insignificant and more studies with standardized BP measurements and validated hypertension diagnoses are warranted.

## Conclusions

While exposure to road traffic noise was associated with systolic BP in subgroups, we were not able to identify a higher risk for self-reported hypertension. Railway noise, on the other hand, seemed positively though insignificant associated with hypertension. More studies are warranted before firm conclusions can be made.

## List of abbreviations

BP: blood pressure; NO_x_: nitrogen oxides; dB(A): decibels; BMI: body mass index; SES: socioeconomic status; IRR: incidence rate ratio; CI: confidence interval.

## Competing interests

The authors declare that they have no competing interests.

## Authors' contributions

MS conceived and designed the study, planned and performed the statistical analyses and drafted the manuscript. MH geokoded addresses and conducted the air pollution calculations. BH, ZA and ORN participated in planning the statistical analyses. JJ collected information on airport and railway noise and RBN digitalized these data. KGL conducted the road traffic noise calculations. AT and KO established the Diet Cancer and Health cohort and provided cohort data. ORN participated in designing the study. All authors participated in interpretation of data, read and commented on the manuscript and approved the final manuscript.
